# Altered Brain Excitability and Increased Anxiety in Mice With Experimental Colitis: Consideration of Hyperalgesia and Sex Differences

**DOI:** 10.3389/fnbeh.2018.00058

**Published:** 2018-04-03

**Authors:** Kewir D. Nyuyki, Nina L. Cluny, Mark G. Swain, Keith A. Sharkey, Quentin J. Pittman

**Affiliations:** ^1^Hotchkiss Brain Institute, Department of Physiology and Pharmacology, Cumming School of Medicine, University of Calgary, Calgary, AB, Canada; ^2^Calvin, Phoebe and Joan Snyder Institute for Chronic Diseases, Cumming School of Medicine, University of Calgary, Calgary, AB, Canada; ^3^Calgary Liver Unit, Division of Gastroenterology, Cumming School of Medicine, University of Calgary, Calgary, AB, Canada

**Keywords:** IBD, colitis, anxiety, pain, diazepam, sex differences

## Abstract

Crohn’s disease (CD) and ulcerative colitis (UC) are incurable lifelong inflammatory bowel diseases (IBD) with a rising worldwide incidence. IBD is characterized by diarrhea, rectal bleeding, severe cramping and weight loss. However, there is a growing evidence that IBD is also associated with anxiety- and depression-related disorders, which further increase the societal burden of these diseases. Given the limited knowledge of central nervous system (CNS) changes in IBD, we investigated CNS-related comorbidities in a mouse model of experimental colitis induced by dextran sulfate sodium (DSS) administration in drinking water for 5 days. In male and female C57BL6J mice, DSS treatment caused increased brain excitability, revealed by a decrease in seizure onset times after intraperitoneal administration of kainic acid. Moreover, both sexes showed increased anxiety-related behavior in the elevated plus-maze (EPM) and open field (OF) paradigms. We assessed somatic pain levels, because they may influence behavioral responses. Only male mice were hyperalgesic when tested with calibrated von Frey hairs and on the hotplate for mechanical and thermal pain sensitivity respectively. Administration of diazepam (DZP; ip, 1 mg/kg) 30 min before EPM rescued the anxious phenotype and improved locomotion, even though it significantly increased thermal sensitivity in both sexes. This indicates that the altered behavioral response is unlikely attributable to an interference with movement due to somatic pain in females. We show that experimental colitis increases CNS excitability in response to administration of kainic acid, and increases anxiety-related behavior as revealed using the EPM and OF tests.

## Introduction

Ulcerative colitis (UC) and Crohn’s disease (CD) are a group of chronic idiopathic inflammatory bowel diseases (IBD) that are characterized by inflammation of the gastrointestinal (GI) tract and severe symptoms including diarrhea, rectal bleeding, severe cramping and weight loss. Behavioral symptoms are commonly present in IBD patients, including reduced emotional well-being (Gralnek et al., 2000), reduced cognitive function (Attree et al., 2003), higher levels of fatigue (Wilson et al., 2004; Graff et al., 2011; Singh et al., 2011), and increased anxiety and depression (Goodhand et al., 2012; Fuller-Thomson et al., 2015; Neuendorf et al., 2016). Interestingly, the prevalence of anxiety and depression are not necessarily related to disease activity (Mikocka-Walus et al., 2016). These psychiatric comorbidities can significantly reduce the quality of life of patients, and contribute significantly to the indirect costs of IBD (Vidal et al., 2008). Despite significant advances in our understanding of the pathogenesis of IBD, it is still unclear how signals from the inflamed gut bring about changes in the central nervous system (CNS) that lead to disease-associated behavioral comorbidities.

Recently, studies using an animal model of IBD induced by dextran sulfate sodium (DSS) administration have demonstrated cognitive impairment, anxiety-like and depressive-like behavior, and decreased social interactions (Bercik et al., 2011; Painsipp et al., 2011; Jain et al., 2015; Emge et al., 2016). In IBD patients, clinical surveys report that affective disorders are generally more prevalent in women than in men (Gorman, 2006). Similarly, a recent study of patients with IBD suggested that women with active disease reported increased anxiety, depression and reduced quality of life (Tribbick et al., 2017). However, most experimental studies to date have used males in order to avoid the intrinsic potential variability associated with female animals. Nonetheless, one study has reported that in the acute DSS model only male mice show an anxious phenotype on the elevated plus-maze (EPM), whereas females show a more depressive-like phenotype in the forced swim test (Painsipp et al., 2011).

Previous studies from our laboratory have shown a microglia-dependent, TNF-α mediated increase in brain excitability (Riazi et al., 2008) associated with altered hippocampal glutamatergic transmission (Riazi et al., 2015) in the 2,4,6-Trinitrobenzenesulfonic acid (TNBS) model of IBD in male rats. Whether this alteration in CNS excitability and behavior also occurs in mice remains to be determined. Moreover, the findings in the TNBS model have not been reproduced in other models of IBD. These are important details, because validation of outcomes in mouse models will enable targeted genetically-based manipulations critical for performing functional studies, and differences between the mechanistic underpinnings of the DSS and TNBS models may have an impact upon CNS signaling (Wirtz and Neurath, 2007). It is also important to know if both males and females show similar susceptibly to altered CNS function and behavior, given the dominance of IBD in women (Betteridge et al., 2013). Finally, given that pain accompanies DSS colitis (Jain et al., 2015; Lapointe et al., 2015), and chronic pain is known to affect both CNS function and behavior (González-Sepúlveda et al., 2016), we also addressed the possible contribution of pain to DSS-associated alterations in behavior.

Using DSS, a well-established animal model of experimental colitis in rodents (Wirtz et al., 2017), we asked if acute DSS colitis in male and female mice: (a) alters CNS excitability; (b) induces an anxiety-like phenotype; and (c) if yes, if the observed anxiety-like phenotype is due to heightened pain sensitivity?

## Materials and Methods

### Animals

Male (20–25 g) and female (18–20 g) C57BL6J mice (7–8 weeks of age, Jackson, Bar Harbor, ME, USA) were housed in groups of four and kept under specific pathogen-free conditions (12:12 light/dark cycle, lights on at 07:00 h, 22°C, 40% humidity, food and water *ad libitum*). One week later (i.e., on day-1: D-1), mice were weighed and randomly assigned to different treatment groups. All experiments were performed during the light phase between 08:00 h and 12:00 noon, in accordance with the Canadian Council on Animal Care regulations and approval from the University of Calgary Animal Care Committee. Unless otherwise stated, day 7 (D7) was the end time point for most experiments, after subjecting animals to behavioral observations. Any mouse that lost more than 20% of its body weight was euthanized. Treatment of animals, methodology, reporting of results and analysis followed the ARRIVE guidelines (Kilkenny et al., 2013). Experimental study groups have been outlined in Table 1.

**Table 1:** Experimental groups and study plan.

	Groups	Day 7	Day 8
Study 1 (46 mice)	Control, DSS (males and females)	Characterization of DSS-induced colitis	
		Assessment of CNS excitability	
Study 2 (59 mice)	Control, DSS (males and females)	Elevated plus-maze (EPM) test	Open field test
Study 3 (24 mice)	DSS + VEH, DSS + DZP (males and females)	DZP intraperitoneal administration followed by EPM test	
Study 4 (126 mice)	Control, DSS, DSS + VEH, DSS + DZP (males and females)	von Frey test, hot plate test, DZP intraperitoneal administration followed by the hot plate test	

### Colitis Induction and Characterization

Experimental mice were given *ad libitum* DSS (40–50 kDa, Affymetrix, Cleveland, OH, USA) in drinking water (2.5% wt/vol) for 5 days (D0 to D5). Control mice drank tap water. On the morning of D5, all DSS-containing bottles were exchanged for tap water for two additional days (until D7), which has been previously determined in preliminary studies to be the peak period of inflammation. During the course of the 7 days, water consumption was monitored and found to be equal between the groups. Body weight and disease activity index were monitored specifically on D0, D2, D5 and D7, and macroscopic colitis damage scores assessed after experiments were completed on D7 or D8.

Colitis disease activity index was based on three measures: body weight loss score, stool consistency and fecal blood, and scored as previously reported (Cooper et al., 1993). Body weight loss scores were determined as the percentage weight loss from the initial body weight on D0 (0 = 0%, 1 = <0 to ≤5%, 2 = >5 to ≤10%, 3 = >10 to ≤15%, 4 = >15%). Stool consistency was scored based on softness/looseness, with normal solid formed stool given a score of 0, soft or sticky stool a 2, and diarrhea (loose and watery stool) a 4. Using hemoccult testing kits from Beckman Coulter, we tested for the presence/absence of fecal blood; a score of 0 (negative hemoccult), 2 (positive hemoccult) or 4 (gross blood) was assigned to each animal tested. The total score per animal was in the range bracket from 0 to 12, where 0 = normal and 12, maximally affected (study 1).

Following behavioral experiments, mice were euthanized using an overdose of isoflurane followed by exsanguination. The colons were resected and examined for macroscopic evidence of colitis and scored for macroscopic damage, as previously described (Cluny et al., 2010). Colon length score was calculated as a percentage of control colon length, with the average control length in males and females being 7 cm and 6.5 cm respectively: (0 = 85%–100%, 1 = 75%–84%, 2 = 65%–74% and 3 < 65%). When present, the length of ulcers was noted (cm). The presence (score = 1) or absence (score = 0) of adhesion, erythema, gross fecal blood and diarrhea was recorded. Body weight loss score was calculated as the % weight loss on D7 vs. D0 using the same parameters as for disease activity index. The sum of the individual macroscopic indices, i.e., body weight loss score, colon thickness (mm), colon length score, length of inflamed colon as % of total length, ulcer length, fecal blood score, diarrhea score, adhesion scores and erythema score were combined to establish the macroscopic damage score for each animal (study 1).

### Experimental Procedures

#### Assessment of CNS Excitability

In order to study the effects of colitis on brain excitability *in vivo*, kainic acid (KA, Nanocs, 25 mg/kg of body weight, 10 ml/kg vol/wt) was administered (intraperitoneally, ip) on the morning of D7 (peak of inflammation). Mice were then individually placed in clear plastic cages, monitored and videotaped for 90 min. The latency to the first behavioral seizure, otherwise called seizure onset time, defined by occurrence of forelimb clonus, rearing and loss of balance were taken as the experimental endpoint as previously described (Minge and Bähring, 2011). Animals were then euthanized for assessment of colonic macroscopic damage score which was later correlated with the seizure onset time (study 1).

#### Behavioral Tests

##### Anxiety-related behavior

The same groups of male and female control or DSS-treated mice were tested on an EPM on D7, and on the following day (D8) an open field (OF) to study the effects of DSS-induced colitis on anxiety-related behavior (study 2).

##### The elevated plus-maze (EPM)

The EPM is used for assessment of anxiety-related behavior in mice (Reber et al., 2007; Nyuyki et al., 2012; Acharjee et al., 2013). Our EPM consisted of two open (6 × 30 cm, 70 lux) and two closed (6 × 30 × 15 cm, 20 lux) arms radiating from a central platform (6 × 6 cm, 55 lux) to form a plus-shaped figure. The maze was elevated 50 cm above the floor. Each mouse was placed on the central platform facing a closed arm and allowed to explore the maze for 5 min. The 5-min test period was recorded by means of a video camera and later analyzed using TopScan^TM^ 2.0 software (Clever Sys Inc., Reston, VA, USA). This allowed for the calculation of the percentage time spent in the arms as well as the total distance traveled, which were deemed measures of anxiety and locomotion respectively. The maze was cleaned thoroughly before each test.

##### The open field (OF)

To screen for anxiety-related behavior as well as to systematically assess novel environment exploration and general locomotor activity (Prut and Belzung, 2003), mice were subjected to an OF test on D8. The OF composed of a blue box (40 × 40 × 40 cm, 40 lux), divided into a central (20 × 20 cm) and peripheral zone. Mice were placed in the central zone and allowed to explore the apparatus for 5 min. The 5-min test period was recorded by means of a video camera and subsequently analyzed automatically using the TopScan^TM^ 2.0 computer software. Anxiety-related behavior was recorded as the percentage time spent in the periphery and central zone, and locomotion represented as the total distance traveled. The apparatus was cleaned thoroughly before each test.

#### Pharmacological Reversal

To verify the presence of an anxious phenotype in DSS-induced colitis, another set of DSS-treated mice were administered diazepam (DZP: Sigma Aldrich^®^, 1 mg/kg, 10 ml/kg, ip) or vehicle (VEH: isotonic saline, B. Braun Medicals Inc., 10 ml/kg, ip) 30 min before exposure to the EPM. DZP is a standard benzodiazepine anxiolytic, commonly employed in behavioral pharmacology as a reference compound (Bert et al., 2001; Pires et al., 2013). The anxiolytic dose used in this study is based on preliminary studies (data not shown), as well as data from other studies (Ohl et al., 2001; study 3).

#### Sensory Function

Pain is a prominent feature of IBD. If animals are in pain, they might move less and this will potentially confound a behavioral phenotype. Thus, to assess generalized somatic pain, we tested different groups of mice for mechanical and thermal nociception on D7. Control and DSS-treated animals were tested with a set of calibrated von Frey hair monofilaments to assess the sensitivity to punctate mechanical stimuli. On the hotplate, the foot withdrawal latency was recorded as a readout for thermal hyperalgesia. In another cohort of DSS-treated animals, we administered DZP (1 mg/kg, 10 ml/kg, ip) or VEH (Saline, 10 ml/kg, ip) and tested for pain on the hotplate 30 min later (study 4).

Mice were placed in clear Plexiglas chambers on an elevated wire mesh screen. Calibrated von Frey hair monofilaments were applied to the plantar surface of each hind paw in the ascending order of bending force (range: 0.04–2.0 g; Olechowski et al., 2013). Each hair was applied five times per paw, and the number of nociceptive responses (vigorous shaking, prolonged lifting, licking or biting of the stimulated paw) was recorded. The monofilament which produced nociceptive responses greater than 60% of the time was taken as the “threshold”. In order to test for thermal hyperalgesia, animals were placed into a Plexiglas rectangular box over a heated plate maintained at 52°C (Ugo Basile; Möser et al., 2011). The latency to the first foot withdrawal, characterized by either hind paw lifting and/or licking of the plantar region was recorded as our behavioral endpoint by an observer blind to treatment. The cut-off limit of 30 s was set to avoid heat damage to foot and the test was performed only once to avoid changes in latency (Lariviere et al., 2002).

### Data Presentation and Statistics

Each data set consists of experiments carried out in DSS vs. control, or DSS + VEH vs. DSS + DZP-treated mice from a minimum of two, and often three cohorts of animals over a period of 2 years. As males and females were tested independently, we did not statistically compare their responses. Data are shown as individual data points as well as mean ± standard error of mean (SEM). Disease activity index and macroscopic damage score are described as median values and represented as mean ± SEM, while acknowledging that these are not parametric values. For statistical analysis, the software package GraphPad Prism 6.0 (La Jolla, CA, USA) was used and differences compared between control and DSS, or DSS + VEH- and DSS + DZP-treated animals by unpaired *t* test for parametric data and the Mann-Whitney U test for non-parametric values. Outliers were identified using the Grubbs’ test, and statistical significance was set at *p* ≤ 0.05.

## Results

### DSS Induces Colitis Severity in Male and Female Mice and Reduces Body Weight Gain

As revealed in the first set of experiments (and typical of all other cohorts in the study-data not shown), both male and female mice treated with DSS lost weight relative to controls (males (*t*_(23)_ = 15.54, *p* < 0.001); females (*t*_(19)_ = 7.471, *p* < 0.001)). Disease activity index was greater for DSS-treated male mice (median = 9.0) than for their respective controls (median = 0.00), *U* = 0.00, *P* < 0.001; as well as DSS-treated females (median = 7.50) vs. controls (median = 0.00), *U* = 0.00, *P* < 0.001 (Mann-Whitney U tests). Furthermore, DSS treatment significantly increased the macroscopic damage score (degree of inflammation) in males (median = 7.50) compared to controls (median = 0.23), *U* = 0.00, *P* < 0.001; likewise in females (median = 5.70) compared to controls (median = 0.19), *U* = 0.00, *P* < 0.001. Colon length (cm) was significantly shorter in DSS-treated males mice (median = 5.8) vs. their respective controls (median = 7.00), *U* = 3.50, *P* < 0.001; as well as DSS-treated females (median = 5.50) compared to controls (median = 6.50), *U* = 13.0, *P* = 0.002. These data indicate that the 2.5% DSS treatment induces all the expected features of colitis (Table 2).

**Table 2:** Characterization of dextran sulfate sodium (DSS)-induced colitis in male and female mice.

	Male		Female	
	Control (*n* = 12)	DSS (*n* = 13)	Control (*n* = 11)	DSS (*n* = 10)
Body weight change (% D0)	3.87 ± 0.59	−15.50 ± 1.10***	3.98 ± 1.0	−8.77 ± 1.36***
Disease activity index	0.67 ± 0.28	8.77 ± 0.72**	0.09 ± 0.09	7.71 ± 0.55***
Macroscopic damage score	0.30 ± 0.08	7.35 ± 0.33***	0.48 ± 0.20	5.70 ± 0.54***
Colon length	7.0 ± 0.14	5.4 ± 0.16***	6.70 ± 0.21	5.40 ± 0.23**

D7 observations reveal a decreased percent body weight gain from D0, increased disease activity index and macroscopic damage score after the administration of 2.5% DSS in drinking water from D0–5. **P < 0.01, ***P < 0.001 denotes a significant difference to the respective control.

### CNS Excitability Is Increased in DSS Colitis

**Figure 1:**
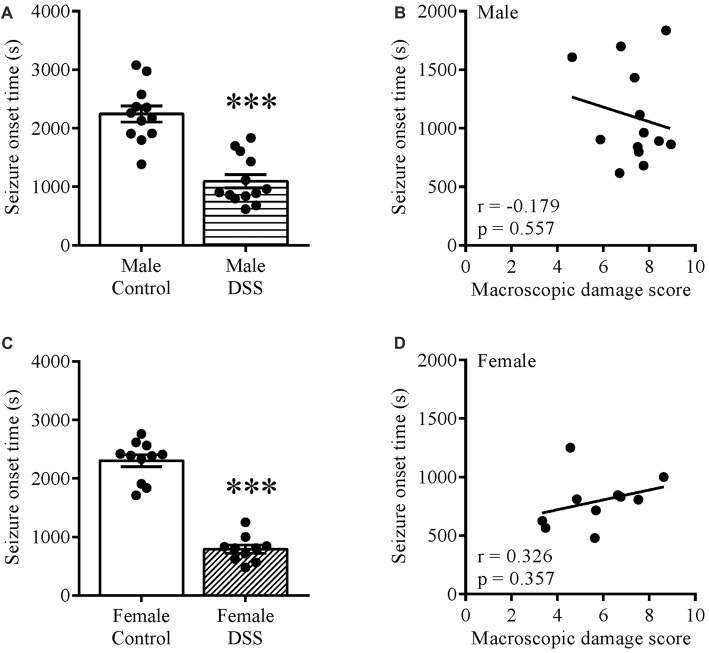
Increased brain excitability in dextran sulfate sodium (DSS) colitis. Five days 2.5% DSS treatment decreased the seizure onset time in both males **(A)** and females **(C)** on day 7 (D7), and did not correlate with the macroscopic damage scores in males **(B)** nor females **(D)**. ***P < 0.001 vs. control mice.

To study the effect of colitis on brain excitability, we injected control and DSS-treated mice with KA ip and noted the seizure onset time for these animals. Statistical analysis revealed a significantly shorter seizure onset time in DSS-treated males (*t*_(23)_ = 6.453, *p* < 0.001; control *n* = 12, DSS *n* = 13; Figure 1A) and females (*t*_(19)_ = 11.980, *p* < 0.001; control *n* = 11, DSS = 10; Figure 1C) vs. their respective controls. Moreover, a Pearson product-moment correlation coefficient was computed to assess the relationship between the seizure onset time and degree of inflammation (macroscopic damage score). Our data revealed no correlation between the two variables in males (*r* = −0.175, *p* = 0.557, *n* = 13; Figure 1B), or in females (*r* = 0.326, *p* = 0.357, *n* = 10; Figure 1D).

### DSS Colitis Increases Anxiety-Related Behavior and Reduces Locomotion on the Elevated Plus-Maze and Open Field

**Figure 2:**
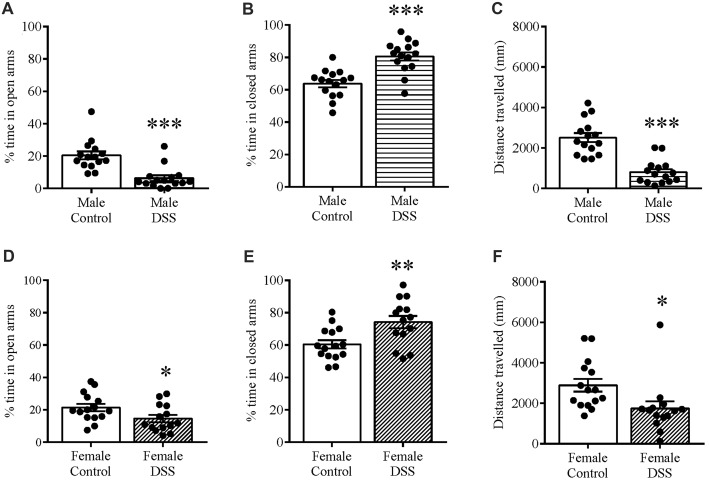
DSS colitis increases anxiety-related behavior and reduces locomotion on the elevated plus-maze (EPM). Male and female control/DSS-treated mice were tested on the EPM on D7. DSS-induced colitis resulted in an anxiety-like phenotype as indicated by reduced percentage time in the unprotected open **(A,D)** and increased time in the closed arm **(B,E)** respectively. Reduced locomotor activity is indicated as the reduced total distance traveled in the maze **(C,F)** in both DSS-treated males and females. ***P < 0.001, **P < 0.01, *P < 0.05 vs. control mice.

When we tested mice using an EPM on D7 to assess anxiety-related behavior, we found that DSS treatment significantly reduced the percentage time spent in the open and unprotected arms of the maze in both males (*t*_(28)_ = 4.730, *p* < 0.001, control *n* = 15, DSS *n* = 15; Figure 2A), and females (*t*_(27)_ = 2.122, *p* < 0.043; control *n* = 14, DSS *n* = 15; Figure 2D), compared with their respective controls, indicating increased anxiety-like behavior. As expected, the percent time in the closed arms was significantly increased in DSS-treated males (*t*_(28)_ = 4.969, *p* < 0.001; Figure 2B) and females (*t*_(27)_ = 3.025, *p* = 0.005; Figure 2E), compared to their respective controls. Locomotion, measured as the total distance traveled in the maze, was less than controls for both DSS-treated males (*t*_(28)_ = 6.326, *p* < 0.001; Figure 2C) and females (*t*_(27)_ = 2.445, *p* = 0.021; Figure 2F).

**Figure 3:**
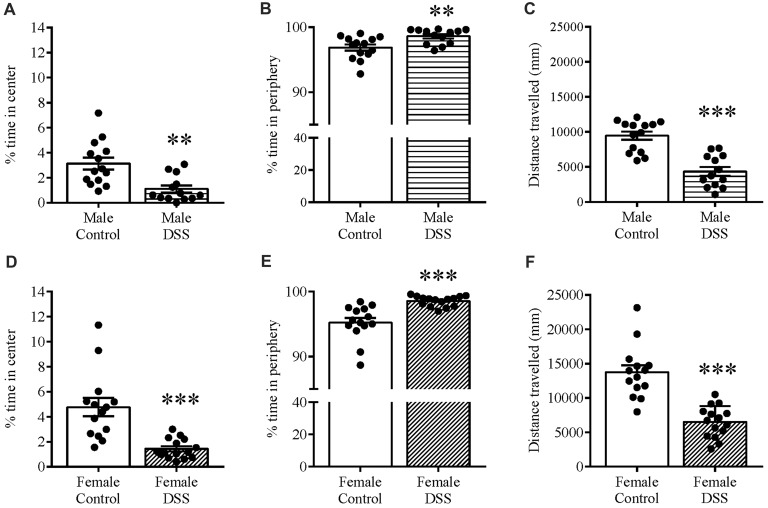
DSS colitis increases anxiety-related behavior and reduces locomotion in the open field (OF). Male and female control/DSS-treated mice, previously tested on the EPM (D7) were subjected to the OF test the next day (D8). In line with previous D7 observations on the EPM, the DSS-induced anxiety-like phenotype remained as indicated by the increased percent time in the peripheral zone of the box **(A,D)**, and reduced center zone percent time **(B,E)** in both sexes. The reduced total distance traveled **(C,F)** was used as a measure of locomotor activity in both males and females. ***P < 0.001, **P < 0.01, vs. control mice.

Anxiety-related behavior was also determined using the OF paradigm, and confirmed the anxious phenotype in these same animals. DSS treatment significantly increased the time spent in the peripheral zone of the OF in both males (*t*_(25)_ = 3.00, *p* = 0.006; control *n* = 14, DSS *n* = 13 Figure 3A) and females (*t*_(27)_ = 4.532, *p* < 0.001; control *n* = 14, DSS *n* = 15; Figure 3D). Furthermore, time spent in the center zone was reduced in both sexes, compared with their respective controls (males: *t*_(25)_ = 3.604, *p* = 0.001; Figure 3B); females: *t*_(27)_ = 4.538, *p* < 0.001; Figure 3E). Reduced locomotor activity was further confirmed as both DSS-treated males and females which moved significantly less than their respective controls (males: *t*_(25)_ = 5.938, *p* < 0.001; Figure 3C); females: *t*_(27)_ = 6.130, *p* < 0.001; Figure 3F).

Note that data are not included from one mouse that jumped out of the EPM during testing, one that lost more than 20% of its bodyweight (D8), and two others that were excluded from the OF analysis as outliers with respect to the Grubb’s test.

### Diazepam Rescues the Anxious Phenotype and Locomotion in DSS-Treated Animals

**Figure 4:**
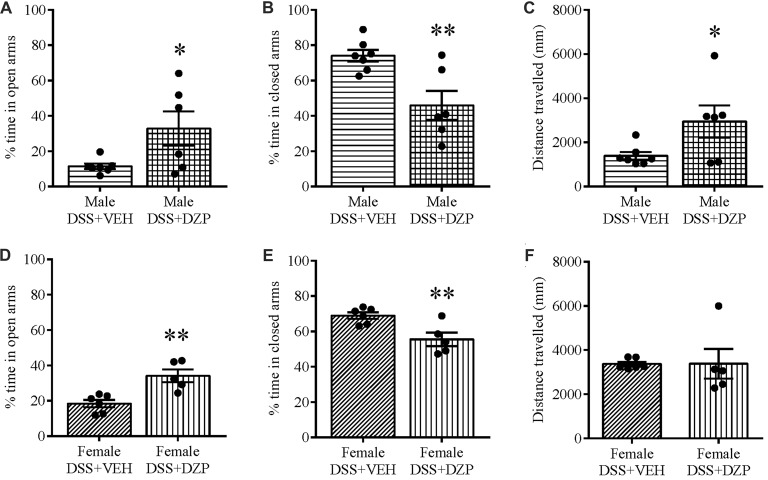
Diazepam (DZP) rescues the anxious phenotype and locomotion in DSS-treated animals. DZP treatment, given 30 min before the EPM test to DSS-treated animals reduced anxiety-related behavior, reflected by the increased percent time spent in the open **(A,D)** and reduced closed arm percent time **(B,E)** in both males **(A,B**) and females **(D,E)**. DZP treatment increased locomotor activity in DSS-treated males **(C)** and rescued **(F)** the decreased locomotion previously seen in DSS-treated females (Figures 2F, 3F). **P < 0.01, *P < 0.05 vs. DSS + VEH mice.

To assess whether reduced open arm behavior in DSS-treated mice was an indication of an anxious phenotype, we administered DZP, an anxiolytic, to cohorts of DSS-treated animals. Administering DZP 30 min before EPM exposure rescued the anxiety-like phenotype in both males and females. DZP + DSS-treated males spent a higher percentage of time in the maze open arms (*t*_(11)_ = 2.358, *p* = 0.038; DSS + VEH *n* = 7, DSS + DZP *n* = 6; Figure 4A) as did females (*t*_(9)_ = 3.944, *p* = 0.003; DSS + VEH *n* = 6, DSS + DZP *n* = 5), when compared to DSS + VEH controls (Figure 4D). On the other hand, the percentage time spent in closed arms was significantly reduced in both males (*t*_(11)_ = 2.365, *p* = 0.006; Figure 4B) and females (*t*_(9)_ = 3.322, *p* = 0.009; Figure 4E). DSS + DZP-treated males moved significantly more than their controls (*t*_(11)_ = 2.231, *p* = 0.05; Figure 4C), while in females, we observed no difference in distance traveled between the DSS + DZP− vs. DSS−VEH controls (*t*_(9)_ = 0.017, *p* = 0.987; Figure 4F).

### Thermal Hyperalgesia in DSS Colitis Is Aggravated by Diazepam

**Figure 5:**
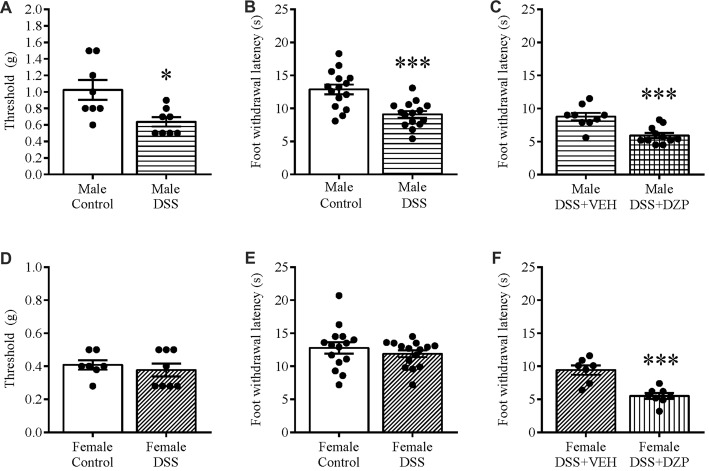
Mechanical and thermal hyperalgesia in DSS colitis: aggravation by DZP. On the morning of D7, separate cohorts of control and DSS-treated mice were tested for mechanical and thermal pain perception by subjecting their paws to calibrated von Frey hairs, or exposing them to the hot plate (52°C) for a maximum of 30 s respectively. DSS treatment reduced withdrawal thresholds **(A)**, and the foot withdrawal latency **(B)** in only males and not females **(D,E)**. A different set of DSS-treated mice were administered VEH or DZP (ip) 30 min before testing. DZP aggravated hyperalgesia by further reducing the foot withdrawal latency in both males **(C)** and females **(F)**. ***P < 0.001, *P < 0.05 vs. control/DSS vehicle mice.

We tested different sets of animals with calibrated von Frey filaments and on the hotplate to investigate whether reduced locomotion and possible unwillingness to venture into the open arm could be due to generalized pain. As one means of assessing this, we evaluated the responsiveness to non-noxious stimuli and noxious heat applied to the paw. Our results revealed that only DSS-treated male mice showed hyperalgesia, presenting a significant decrease in withdrawal thresholds to punctate mechanical stimulation (*t*_(14)_ = 2.909, *p* = 0.011; control *n* = 8, DSS *n* = 8; Figure 5A), and reduced foot withdrawal latency (*t*_(28)_ = 4.243, *p* < 0.001; control *n* = 15, DSS *n* = 15; Figure 5B), that was not observed in females (*t*_(13)_ = 0.631, *p* = 0.539; control = 15, DSS *n* = 15; Figure 5D); (*t*_(28)_ = 0.887, *p* = 0.382; control = 15, DSS *n* = 15; Figure 5E), when compared with their respective controls. As in the previous experiment, DZP treatment reversed the anxious phenotype but also improved locomotion in both sexes (in Figure 4), therefore we asked if the DZP was merely reversing pain behavior, rather than anxious behavior. Thus, we tested, in another cohort of animals, the effect of DZP on foot withdrawal latency in mice with DSS colitis. This study revealed heightened thermal pain perception in DZP + DSS-treated males (*t*_(18)_ = 4.280, *p* < 0.001; DSS + VEH *n* = 9, DSS + DZP *n* = 11; Figure 5C), and females (*t*_(13)_ = 4.867, *p* < 0.001; DSS + VEH *n* = 7, DSS + DZP *n* = 8; Figure 5F), vs. their respective DSS + VEH controls.

## Discussion

Our studies are the first to present behavioral findings regarding anxiety, pain and CNS excitability in male and female mice with acute DSS-induced colitis. We show that experimental colitis increases CNS excitability in response to administration of KA, and increases anxiety-related behavior as revealed on the EPM and OF. There was decreased locomotion in these tests for both males and females with colitis. DZP rescued the DSS-induced anxious phenotype and improved locomotion in both sexes. We also found increased mechanical and thermal pain sensitivity in males but not in females, and DZP significantly increased the thermal pain sensitivity after DSS in both males and females.

We used the DSS colitis model to study the behavioral comorbidities associated with IBD because it is one of the most commonly used, reliable and reproducible models of experimental colitis (Chassaing et al., 2014; Wirtz et al., 2017). Although the exact mechanism(s) by which DSS causes colitis is (are) not fully understood, it is clear that DSS, which has a molecular weight of 40–50 kDa, does not cross the blood brain barrier. Therefore, the behavioral effects described in the current study are likely due to DSS interactions with the intestinal epithelium, as previously suggested (Chassaing et al., 2014), which initiates gut-brain communication (Keightley et al., 2015). Several studies have previously reported altered anxiety-like behavior after DSS exposure, and the present findings confirm these reports (Bercik et al., 2011; Painsipp et al., 2011; Hassan et al., 2014; Jain et al., 2015; Emge et al., 2016). However, our study is novel in that it reports this phenotype in both sexes. Interestingly, this is a finding that was not evident in one of the studies that used both sexes, as they reported anxiety- and depressive-like behavior in males and females respectively (Painsipp et al., 2011). While we did not directly compare responses between males and females, inspection of the data suggests that the magnitude of the responses in both sexes was similar. Although a recent clinical study reported that female patients with active CD showed a higher degree of illness perception and consequently, more psychological distress (Tribbick et al., 2017), others confirm a high prevalence of anxiety and depression in both male and female IBD patients (Goodhand et al., 2012; Sajadinejad et al., 2012; Fuller-Thomson et al., 2015; Neuendorf et al., 2016); findings that were independent of the severity of IBD disease activity (Mikocka-Walus et al., 2016). In this regard, it is important to point out that our anxiety-like behavior is coincident with the acute disease activity and does not necessarily represent an ongoing anxiety disorder. Future studies will address this by examining anxiety phenotype following resolution of the acute colitis.

We acknowledge that reduced locomotion in both sexes on the EPM and in the OF could limit the interpretation of our anxiety phenotype. Locomotor activity of individual animals on the EPM and OF is determined by their reaction to novelty, and reduced locomotion may in part, be a consequence of enhanced anxiety-like behavior. However, IBD is known to be accompanied by chronic abdominal pain (Bielefeldt et al., 2011), a finding also present in rodent models of IBD. Colitis is associated with dorsal root ganglion hyper-excitability (Lomax et al., 2007) and increased visceral pain sensitivity (Verma-Gandhu et al., 2007; Lapointe et al., 2015), and, as we show here, mechanical and thermal hyperalgesia using von Frey monifilaments and in the hot plate test respectively. However, with the use of an anxiolytic (DZP) that has been validated for behavioral anxiety tests (Mohammad et al., 2016), we reversed the DSS-induced anxiety-like phenotype on the EPM. This finding suggests that the behavioral responses we observed may be indicative of an anxious phenotype in mice with colitis. We showed that, at least with responses to thermal pain, DZP actually increased pain sensitivity. Similar pro-nociceptive effects of DZP have been previously reported in mice (Rosland et al., 1987; Rosland and Hole, 1990; Pakulska and Czarnecka, 2001), and it may be due to a reduction of stress-induced analgesia. It would be premature to surmise that DZP also alters visceral pain and further studies on the effects of DZP on mechanical and visceral hyperalgesia will be more conclusive. In fact, as inflammatory pain, in itself, can induce anxiety (Parent et al., 2012), it is possible that the presence of anxiety may be due wholly or in part to the visceral pain associated with DSS-induced colitis.

The responses we observed in DSS-treated mice may represent a true anxious phenotype. As both males and females showed increased anxiety-like behavior, whereas only males showed a hyperalgesic phenotype, it is possible that it may occur independently of pain and further studies will be required to assess this. However, if thermal hyperalgesia occurs concurrently with visceral pain in acute DSS colitis as we and others (Jain et al., 2015) have shown, this would suggest that, at least for females, the preference for the closed arm of the EPM and the periphery of the OF was not due to pain induced immobility, as they showed neither mechanical nor thermal hyperalgesia. This finding is interesting as, in the clinical milieu, reports highlight the substantially greater prevalence of many clinical pain conditions in women vs. men, and growing evidence for sex differences in sensitivity to experimental pain and to analgesics (Robinson et al., 2001; Greenspan et al., 2007).

Our findings of a reversal of the anxiety-like phenotype in DSS colitis complements that of Bercik et al. (2011), who were able to completely reverse the anxious phenotype by vagotomy in DSS-treated mice in the step down test, suggesting that this is a vagally mediated pain independent-effect. This suggests that the mechanisms associated with these phenotypes can be uncoupled, supplementing our findings that the anxiety-like phenotype may not be due to reduced locomotion, and that, at least in females, is independent of increased mechanical and thermal pain sensitivity.

Moreover, we show here that hyperalgesia is not simply restricted to the gut, as DSS treated male mice showed increased mechanical and thermal sensitivity to von Frey monofilaments and in the hot plate test respectively. The mechanism by which sensory afferents from the skin become sensitized by gut inflammation is not known, but is in accord with previous studies showing that there is cross- talk among afferent nerve populations in colitis, as rats with inflamed colons have sensitized urinary bladder afferents (Qin et al., 2005; Malykhina et al., 2006). The reason that there are sex-dependent effects here is not currently known, but there is evidence in the literature for sexual dimorphism in mechanisms underlying pain sensitivity (Mapplebeck et al., 2016).

The prevalence of behavioral comorbidities in IBD pinpoints CNS involvement, as previously reviewed by us (Nyuyki and Pittman, 2015). In the present study, we have shown that DSS-treated male and female mice display decreased seizure onset times in response to administration of KA. Interestingly, decreased seizure onset times can be interpreted as increased excitability, which in this study did not correlate with the degree of colonic damage (macroscopic damage score) in either males or females. That central neuronal excitability is altered in an experimental model of colitis is congruent with our previous findings in TNBS-induced colitis in male rats. As these changes persist when brain tissue (hippocampus) is excised, this cannot be due to altered convulsant distribution, changes in metabolism or body temperature (Riazi et al., 2008). It is important to note that the increased seizure susceptibility should not be interpreted as indicative of a pro-seizure or pro-epilepsy phenotype; in this study, it is simply used as an assay for an altered brain state. Inflammation-induced changes in synaptic and membrane properties like enhanced AMPA- and NMDA-mediated synaptic currents, and Schaffer collateral-induced excitatory field potentials in CA1 stratum radiatum amongst others as previously reported by us (Riazi et al., 2015) could be responsible for the altered CNS excitability. The pathways by which information from the inflamed colon communicates to the brain likely includes circulating immune cells and inflammatory mediators that communicate directly via the brain endothelium, or via afferent vagal or spinal afferent nerves that transmit information to the brain (Goehler et al., 2000, 2005; Schiltz and Sawchenko, 2002; Inoue et al., 2008). Once IBD develops, the unpredictability, uncertainty and chronic course of the disease can cause a wide range of psychological and interpersonal concerns to patients. The presence of psychiatric comorbidities including anxiety, depression, fatigue and cognitive impairment in IBD patients have undoubtedly led to increased reports of reduced quality of life and general well-being (Guthrie et al., 2002; Nordin et al., 2002). In the clinical setting, it remains unclear if IBD is a risk or causative factor for these psychiatric comorbidities. Our study lays the groundwork for mechanistic explorations related to how the inflamed gut communicates to the brain and how specific neuronal and synaptic properties are altered in colitis and are different in the context of different sexes.

## Author Contributions

KDN and QJP designed the study. KDN and NLC performed experiments and analyzed data. KDN, NLC and QJP wrote the manuscript. NLC and KAS edited the manuscript. MGS, KAS and QJP supervised the project and edited the manuscript.

## Conflict of Interest Statement

The authors declare that the research was conducted in the absence of any commercial or financial relationships that could be construed as a potential conflict of interest. The reviewer DLV and handling Editor declared their shared affiliation.

## References

[B1] Acharjee S. Nayani N. Tsutsui M. Hill M. N. Ousman S. S. Pittman Q. J. (2013). Altered cognitive-emotional behavior in early experimental autoimmune encephalitis—cytokine and hormonal correlates. Brain Behav. Immun. 33, 164–172. 10.1016/j.bbi.2013.07.003 23886782

[B2] Attree E. A. Dancey C. P. Keeling D. Wilson C. (2003). Cognitive function in people with chronic illness: inflammatory bowel disease and irritable bowel syndrome. Appl. Neuropsychol. 10, 96–104. 10.1207/s15324826an1002_05 12788684

[B3] Bercik P. Park A. J. Sinclair D. Khoshdel A. Lu J. Huang X.. (2011). The anxiolytic effect of Bifidobacterium longum NCC3001 involves vagal pathways for gut-brain communication. Neurogastroenterol. Motil. 23, 1132–1139. 10.1111/j.1365-2982.2011.01796.x 21988661 PMC3413724

[B4] Bert B. Fink H. Sohr R. Rex A. (2001). Different effects of diazepam in Fischer rats and two stocks of Wistar rats in tests of anxiety. Pharmacol. Biochem. Behav. 70, 411–420. 10.1016/s0091-3057(01)00629-3 11701214

[B5] Betteridge J. D. Armbruster S. P. Maydonovitch C. Veerappan G. R. (2013). Inflammatory bowel disease prevalence by age, gender, race, and geographic location in the U.S. Military health care population. Inflamm. Bowel Dis. 19, 1421–1427. 10.1097/MIB.0b013e318281334d 23518811

[B6] Bielefeldt K. Davis B. Binion D. (2011). Pain and inflammatory bowel disease. Inflamm. Bowel Dis. 15, 778–788. 10.1002/ibd.20848 19130619 PMC3180862

[B7] Chassaing B. Aitken J. D. Malleshappa M. Vijay-Kumar M. (2014). Dextran sulfate sodium (DSS)-induced colitis in mice. Curr. Protoc. Immunol. 104:Unit 15.25. 10.1002/0471142735.im1525s104 24510619 PMC3980572

[B8] Cluny N. L. Keenan C. M. Duncan M. Fox A. Lutz B. Sharkey K. A. (2010). Naphthalen-1-yl-(4-pentyloxynaphthalen-1-yl)methanone (SAB378), a peripherally restricted cannabinoid CB1/CB2 receptor agonist, inhibits gastrointestinal motility but has no effect on experimental colitis in mice. J. Pharmacol. Exp. Ther. 334, 973–980. 10.1124/jpet.110.169946 20571060

[B9] Cooper H. S. Murthy S. N. Shah R. S. Sedergran D. J. (1993). Clinicopathologic study of dextran sulfate sodium experimental murine colitis. Lab. Invest. 69, 238–249. 8350599

[B10] Emge J. R. Huynh K. Miller E. N. Kaur M. Reardon C. Barrett K. E.. (2016). Modulation of the microbiota-gut-brain axis by probiotics in a murine model of inflammatory bowel disease. Am. J. Physiol. Gastrointest. Liver Physiol. 310, G989–G998. 10.1152/ajpgi.00086.2016 27056723

[B11] Fuller-Thomson E. Lateef R. Sulman J. (2015). Robust association between inflammatory bowel disease and generalized anxiety disorder. Inflamm. Bowel Dis. 21, 2341–2348. 10.1097/MIB.0000000000000518 26218145

[B12] Goehler L. E. Gaykema R. P. A. Opitz N. Reddaway R. Badr N. Lyte M. (2005). Activation in vagal afferents and central autonomic pathways: early responses to intestinal infection with Campylobacter jejuni. Brain Behav. Immun. 19, 334–344. 10.1016/j.bbi.2004.09.002 15944073

[B13] Goehler L. E. Gaykema R. P. Hansen M. K. Anderson K. Maier S. F. Watkins L. R. (2000). Vagal immune-to-brain communication: a visceral chemosensory pathway. Auton. Neurosci. 85, 49–59. 10.1016/s1566-0702(00)00219-8 11189026

[B14] González-Sepúlveda M. Pozo O. J. Marcos J. Valverde O. (2016). Chronic pain causes a persistent anxiety state leading to increased ethanol intake in CD1 mice. J. Psychopharmacol. Oxford 30, 188–203. 10.1177/0269881115622238 26681793

[B15] Goodhand J. R. Wahed M. Mawdsley J. E. Farmer A. D. Aziz Q. Rampton D. S. (2012). Mood disorders in inflammatory bowel disease: relation to diagnosis, disease activity, perceived stress, and other factors. Inflamm. Bowel Dis. 18, 2301–2309. 10.1002/ibd.22916 22359369

[B102] Gorman J. M. (2006). Gender differences in depression and response to psychotropic medication. Gend. Med. 3, 93–109. 10.1016/S1550-8579(06)80199-3 16860269

[B16] Graff L. A. Vincent N. Walker J. R. Clara I. Carr R. Ediger J.. (2011). A population-based study of fatigue and sleep difficulties in inflammatory bowel disease. Inflamm. Bowel Dis. 17, 1882–1889. 10.1002/ibd.21580 21830266

[B17] Gralnek I. M. Hays R. D. Kilbourne A. Naliboff B. Mayer E. A. (2000). The impact of irritable bowel syndrome on health-related quality of life. Gastroenterology 119, 654–660. 10.1053/gast.2000.16484 10982758

[B103] Greenspan J. D. Craft R. M. LeResche L. Arendt-Nielsen L. Berkley K. J. Fillingim R. B.. (2007). Studying sex and gender differences in pain and analgesia: a consensus report. Pain 132, S26–S45. 10.1016/j.pain.2007.10.014 17964077 PMC2823483

[B104] Guthrie E. Jackson J. Shaffer J. Thompson D. Tomenson B. Creed F. (2002). Psychological disorder and severity of inflammatory bowel disease predict health-related quality of life in ulcerative colitis and Crohn’s disease. Am. J. Gastroenterol. 97, 1994–1999. 10.1111/j.1572-0241.2002.05842.x 12190166

[B105] Hassan A. M. Jain P. Reichmann F. Mayerhofer R. Farzi A. Schuligoi R.. (2014). Repeated predictable stress causes resilience against colitis-induced behavioral changes in mice. Front. Behav. Neurosci. 8:386. 10.3389/fnbeh.2014.00386 25414650 PMC4222228

[B18] Inoue W. Somay G. Poole S. Luheshi G. N. (2008). Immune-to-brain signaling and central prostaglandin E2 synthesis in fasted rats with altered lipopolysaccharide-induced fever. Am. J. Physiol. Regul. Integr. Comp. Physiol. 295, R133–R143. 10.1152/ajpregu.90335.2008 18480240 PMC2494823

[B19] Jain P. Hassan A. M. Koyani C. N. Mayerhofer R. Reichmann F. Farzi A.. (2015). Behavioral and molecular processing of visceral pain in the brain of mice: impact of colitis and psychological stress. Front. Behav. Neurosci. 9:177. 10.3389/fnbeh.2015.00177 26217204 PMC4498125

[B20] Keightley P. C. Koloski N. A. Talley N. J. (2015). Pathways in gut-brain communication: evidence for distinct gut-to-brain and brain-to-gut syndromes. Aust. New Zeal. J. Psychiatry 49, 207–214. 10.1177/0004867415569801 25710826

[B21] Kilkenny C. Browne W. J. Cuthill I. C. Emerson M. Altman D. G. (2013). Improving bioscience research reporting: the ARRIVE guidelines for reporting animal research. Animals 4, 35–44. 10.3390/ani4010035 22424462

[B22] Lapointe T. K. Basso L. Iftinca M. C. Flynn R. Chapman K. Dietrich G.. (2015). TRPV1 sensitization mediates postinflammatory visceral pain following acute colitis. Am. J. Physiol. Gastrointest. Liver Physiol. 309, G87–G99. 10.1152/ajpgi.00421.2014 26021808

[B23] Lariviere W. R. Wilson S. G. Laughlin T. M. Kokayeff A. West E. E. Adhikari S. M.. (2002). Heritability of nociception. III. Genetic relationships among commonly used assays of nociception and hypersensitivity. Pain 97, 75–86. 10.1016/s0304-3959(01)00492-4 12031781

[B24] Lomax A. E. O ’reilly M. Neshat S. Vanner S. J. (2007). Sympathetic vasoconstrictor regulation of mouse colonic submucosal arterioles is altered in experimental colitis. J. Physiol. 5832, 719–730. 10.1113/jphysiol.2007.136838 17615098 PMC2277024

[B25] Malykhina A. P. Qin C. Greenwood-Van Meerveld B. Foreman R. D. Lupu F. Akbarali H. I. (2006). Hyperexcitability of convergent colon and bladder dorsal root ganglion neurons after colonic inflammation: mechanism for pelvic organ cross-talk. Neurogastroenterol. Motil. 18, 936–948. 10.1111/j.1365-2982.2006.00807.x 16961697

[B26] Mapplebeck J. C. S. Beggs S. Salter M. W. (2016). Sex differences in pain: a tale of two immune cells. Pain 157, S2–S6. 10.1097/j.pain.0000000000000389 26785152

[B27] Mikocka-Walus A. Pittet V. Rossel J.-B. von Känel R. Anderegg C. Bauerfeind P.. (2016). Symptoms of depression and anxiety are independently associated with clinical recurrence of inflammatory bowel disease. Clin. Gastroenterol. Hepatol. 14, 829.e1–835.e1. 10.1016/j.cgh.2015.12.045 26820402

[B28] Minge D. Bähring R. (2011). Acute alterations of somatodendritic action potential dynamics in hippocampal ca1 pyramidal cells after kainate-induced status epilepticus in mice. PLoS One 6:e26664. 10.1371/journal.pone.0026664 22039527 PMC3200351

[B29] Mohammad F. Ho J. Woo J. H. Lim C. L. Jun D. Poon J.. (2016). Concordance and incongruence in preclinical anxiety models: systematic review and meta-analyses. Neurosci. Biobehav. Rev. 68, 504–529. 10.1016/j.neubiorev.2016.04.011 27328783

[B30] Möser C. V. Kynast K. Baatz K. Russe O. Q. Ferreirós N. Costiuk H.. (2011). The protein kinase IKKε is a potential target for the treatment of inflammatory hyperalgesia. J. Immunol. 187, 2617–2625. 10.4049/jimmunol.1004088 21813779

[B31] Neuendorf R. Harding A. Stello N. Hanes D. Wahbeh H. (2016). Depression and anxiety in patients with inflammatory bowel disease: a systematic review. J. Psychosom. Res. 87, 70–80. 10.1016/j.jpsychores.2016.06.001 27411754

[B101] Nordin K. Påhlman L. Larsson K. Sundberg-Hjelm M. Lööf L. (2002). Health-related quality of life and psychological distress in a population-based sample of Swedish patients with inflammatory bowel disease. Scand. J. Gastroenterol. 37, 450–457. 10.1080/003655202317316097 11989837

[B33] Nyuyki K. D. Maloumby R. Reber S. O. Neumann I. D. (2012). Comparison of corticosterone responses to acute stressors: chronic jugular vein versus trunk blood samples in mice. Stress 15, 618–626. 10.3109/10253890.2012.655348 22251167

[B32] Nyuyki K. D. Pittman Q. J. (2015). Toward a better understanding of the central consequences of intestinal inflammation. Ann. N Y Acad. Sci. 1351, 149–154. 10.1111/nyas.12935 26378439

[B34] Ohl F. Sillaber I. Binder E. Keck M. E. Holsboer F. (2001). Differential analysis of behavior and diazepam-induced alterations in C57BL/6N and BALB/c mice using the modified hole board test. J. Psychiatr. Res. 35, 147–154. 10.1016/s0022-3956(01)00017-6 11461710

[B35] Olechowski C. J. Tenorio G. Sauve Y. Kerr B. J. (2013). Changes in nociceptive sensitivity and object recognition in experimental autoimmune encephalomyelitis (EAE). Exp. Neurol. 241, 113–121. 10.1016/j.expneurol.2012.12.012 23291347

[B36] Painsipp E. Herzog H. Sperk G. Holzer P. (2011). Sex-dependent control of murine emotional-affective behaviour in health and colitis by peptide YY and neuropeptide y. Br. J. Pharmacol. 163, 1302–1314. 10.1111/j.1476-5381.2011.01326.x 21410462 PMC3144542

[B37] Pakulska W. Czarnecka E. (2001). Effect of diazepam and midazolam on the antinociceptive effect of morphine, metamizol and indomethacin in mice. Pharmazie 56, 89–91. 11210678

[B38] Parent A. J. Beaudet N. Beaudry H. Bergeron J. Bérubé P. Drolet G.. (2012). Increased anxiety-like behaviors in rats experiencing chronic inflammatory pain. Behav. Brain Res. 229, 160–167. 10.1016/j.bbr.2012.01.001 22245257 PMC3848972

[B39] Pires L. F. Costa L. M. Silva O. A. de Almeida A. A. C. Cerqueira G. S. de Sousa D. P.. (2013). Anxiolytic-like effects of carvacryl acetate, a derivative of carvacrol, in mice. Pharmacol. Biochem. Behav. 112, 42–48. 10.1016/j.pbb.2013.09.001 24036473

[B40] Prut L. Belzung C. (2003). The open field as a paradigm to measure the effects of drugs on anxiety-like behaviors: a review. Eur. J. Pharmacol. 463, 3–33. 10.1016/s0014-2999(03)01272-x 12600700

[B41] Qin C. Malykhina A. P. Akbarali H. I. Foreman R. D. (2005). Cross-organ sensitization of lumbosacral spinal neurons receiving urinary bladder input in rats with inflamed colon. Gastroenterology 129, 1967–1978. 10.1053/j.gastro.2005.09.013 16344065

[B42] Reber S. O. Birkeneder L. Veenema A. H. Obermeier F. Falk W. Straub R. H.. (2007). Adrenal insufficiency and colonic inflammation after a novel chronic psycho-social stress paradigm in mice: implications and mechanisms. Endocrinology 148, 670–682. 10.1210/en.2006-0983 17110427

[B43] Riazi K. Galic M. A. Kentner A. C. Reid A. Y. Sharkey K. A. Pittman Q. J. (2015). Microglia-dependent alteration of glutamatergic synaptic transmission and plasticity in the hippocampus during peripheral inflammation. J. Neurosci. 35, 4942–4952. 10.1523/JNEUROSCI.4485-14.2015 25810524 PMC6705378

[B44] Riazi K. Galic M. A. Kuzmiski J. B. Ho W. Sharkey K. A. Pittman Q. J. (2008). Microglial activation and TNFα production mediate altered CNS excitability following peripheral inflammation. Proc. Natl. Acad. Sci. U S A 105, 17151–17156. 10.1073/pnas.0806682105 18955701 PMC2579393

[B106] Robinson M. E. Riley J. L. III. Myers C. D. Papas R. K. Wise E. A. Waxenberg L. B.. (2001). Gender role expectations of pain: relationship to sex differences in pain. J. Pain 2, 251–257. 10.1054/jpai.2001.24551 14622803

[B45] Rosland J. H. Hole K. (1990). 1,4-benzodiazepines antagonize opiate-induced antinociception in mice. Anesth. Analg. 71, 242–248. 10.1213/00000539-199009000-00005 2393107

[B46] Rosland J. H. Hunskaar S. Hole K. (1987). The effect of diazepam on nociception in mice. Pharmacol. Toxicol. 61, 111–115. 10.1111/j.1600-0773.1987.tb01786.x 3118353

[B47] Sajadinejad M. S. Asgari K. Molavi H. Kalantari M. Adibi P. (2012). Psychological issues in inflammatory bowel disease: an overview. Gastroenterol. Res. Pract. 2012:106502. 10.1155/2012/106502 22778720 PMC3388477

[B48] Schiltz J. C. Sawchenko P. E. (2002). Distinct brain vascular cell types manifest inducible cyclooxygenase expression as a function of the strength and nature of immune insults. J. Neurosci. 22, 5606–5618. 12097512 10.1523/JNEUROSCI.22-13-05606.2002 PMC6758199

[B49] Singh S. Blanchard A. Walker J. R. Graff L. A. Miller N. Bernstein C. N. (2011). Common symptoms and stressors among individuals with inflammatory bowel diseases. Clin. Gastroenterol. Hepatol. 9, 769–775. 10.1016/j.cgh.2011.05.016 21645640

[B50] Tribbick D. Salzberg M. Connell W. Macrae F. Kamm M. Bates G.. (2017). Differences across illness perceptions in inflammatory bowel disease and their relationships to psychological distress and quality of life. Gastroenterol. Nurs. 40, 291–299. 10.1097/SGA.0000000000000225 28746114

[B51] Verma-Gandhu M. Verdu E. F. Bercik P. Blennerhassett P. A. Al-Mutawaly N. Ghia J.-E.. (2007). Visceral pain perception is determined by the duration of colitis and associated neuropeptide expression in the mouse. Gut 56, 358–364. 10.1136/gut.2006.100016 17018864 PMC1856796

[B52] Vidal A. Gómez-Gil E. Sans M. Portella M. J. Salamero M. Piqué J. M.. (2008). Health-related quality of life in inflammatory bowel disease patients: the role of psychopathology and personality. Inflamm. Bowel Dis. 14, 977–983. 10.1002/ibd.20388 18275078

[B53] Wilson A. Reyes E. Ofman J. (2004). Prevalence and outcomes of anemia in inflammatory bowel disease: a systematic review of the literature. Am. J. Med. 116, 44S–49S. 10.1016/j.amjmed.2003.12.011 15050885

[B54] Wirtz S. Neurath M. F. (2007). Mouse models of inflammatory bowel disease. Adv. Drug Deliv. Rev. 59, 1073–1083. 10.1016/j.addr.2007.07.003 17825455

[B55] Wirtz S. Popp V. Kindermann M. Gerlach K. Weigmann B. Fichtner-Feigl S.. (2017). Chemically induced mouse models of acute and chronic intestinal inflammation. Nat. Protoc. 12, 1295–1309. 10.1038/nprot.2017.044 28569761

